# Using Step Size and Lower Limb Segment Orientation from Multiple Low-Cost Wearable Inertial/Magnetic Sensors for Pedestrian Navigation

**DOI:** 10.3390/s19143140

**Published:** 2019-07-17

**Authors:** Chandra Tjhai, Kyle O’Keefe

**Affiliations:** Position, Location, and Navigation (PLAN) Group, Department of Geomatics Engineering, Schulich School of Engineering, University of Calgary, 2500 University Drive, N.W., Calgary, AB T2N 1N4, Canada

**Keywords:** forward kinematics, pitch angle, skeletal model, step size, step length, stride length, wearable sensors, wearable multi-sensor system

## Abstract

This paper demonstrates the use of multiple low-cost inertial/magnetic sensors as a pedestrian navigation system for indoor positioning. This research looks at the problem of pedestrian navigation in a practical manner by investigating dead-reckoning methods using low-cost sensors. This work uses the estimated sensor orientation angles to compute the step size from the kinematics of a skeletal model. The orientations of limbs are represented by the tilt angles estimated from the inertial measurements, especially the pitch angle. In addition, different step size estimation methods are compared. A sensor data logging system is developed in order to record all motion data from every limb segment using a single platform and similar types of sensors. A skeletal model of five segments is chosen to model the forward kinematics of the lower limbs. A treadmill walk experiment with an optical motion capture system is conducted for algorithm evaluation. The mean error of the estimated orientation angles of the limbs is less than 6 degrees. The results show that the step length mean error is 3.2 cm, the left stride length mean error is 12.5 cm, and the right stride length mean error is 9 cm. The expected positioning error is less than 5% of the total distance travelled.

## 1. Introduction

Development of micro-electromechanical systems (MEMS) has enabled miniaturization of many sensors. The common sensors used in navigation applications are accelerometers, gyroscopes, magnetometers, and barometers. MEMS technology has enabled these sensors to be miniaturized to the level of an integrated circuit chip with very low weight, power consumption, and cost and these devices are commonly found as embedded sensors inside smartphones and fitness trackers.

Over the past decade, pedestrian navigation systems have received much attention due to the emergence of MEMS sensors, which have allowed the pedestrian to carry inertial measurement units. An inertial sensor usually consists of an accelerometer triad and a gyroscope triad. In the early development of pedestrian navigation systems, the Defence Advanced Research Project Agency (DARPA) demonstrated a Small Unit Operations/Situation Awareness System (SUO/SAS) for tracking military personnel [1]. This SUO/SAS system was a bulky backpack device and consisted of a Global Positioning System (GPS) receiver, a tactical grade MEMS inertial measurement unit (IMU), a barometer, and a compass. A high performance pedestrian navigation systems is not only needed by the military but also by first responders to perform firefighting and search and rescue operations. Rantakokko et al. [2] describe the requirements of pedestrian navigation systems for military personnel and first responders. These authors emphasize that the system should be lightweight, small size, inexpensive (∼1000 US dollars), power efficient, and provide metre-level accuracy for seamless transitions between outdoor and indoor environments.

In the current state-of-the-art technology, it is not difficult to a develop low-cost, lightweight, and small size navigation system. The challenge lies in the trade-off between accuracy and total cost. The advantages of IMUs are that they are a self-contained system and cannot be jammed by radio signals. However, inertial sensors usually suffer from error accumulation due to integration of uncorrected biases [3] and low-cost MEMS sensors usually have larger noise levels than other grades of inertial sensors [4]. The noise level of MEMS sensors is inversely related with the cost of the device. To mitigate this issue, researchers have performed integration of inertial systems with other aiding systems like Global Navigation Satellite Systems (GNSS) for outdoor environments [5] and wireless sensors for indoor applications [6]. GNSS-based positioning is often ineffective, unreliable, or unavailable for indoor users due to signal attenuation, multipath, and blockages [7].

In the field of pedestrian navigation for civilian use, one common research platform is a smartphone, because this device has been equipped with inertial sensors, a GNSS receiver, and wireless connectivity. Zhuang et al. [6] implemented the integration of an inertial navigation system (INS) with wireless network (WiFi) fingerprinting on a smartphone with a reported accuracy of the integrated system of less than 5 m for indoor environments. A more smartphone-based navigation algorithm was reported by Ebner et al. [8]. The authors integrated inertial, barometer, WiFi, and iBeacons to perform three-dimensional localization in indoor environments. However, these methods are too dependent on building infrastructure where WiFi and floor plan maps are not always available.

Many inertial-based pedestrian navigation algorithms have been developed and tested by researchers. These inertial-based algorithms utilize the dead-reckoning method in computing the navigation solution. There are two types of inertial-based algorithms: A strapdown system and a step-heading system [9,10]. The former method uses mathematical computation to extract a positioning solution from accelerations and orientations in three dimensional space. Meanwhile, the latter method uses the inertial sensor to compute step size and direction of motion, then combines these two parameters to obtain a relative navigation solution. The step-heading dead-reckoning algorithm reduces the positioning error from proportional to time cubed (in the strapdown case) to a small fraction of total distance travelled.

Pedestrian navigation systems can also be classified based on the location of the inertial sensors on the human body. In the early development of pedestrian dead-reckoning (PDR) algorithms, Foxlin [11] reported a pedestrian tracking using a foot-mounted inertial sensor and zero-velocity constraint. The reported results showed that the Kalman filter-based positioning error was 0.3% of the 118.5 m walking path. Meanwhile, Godha and Lachapelle [12] demonstrated integration of a GNSS receiver, foot-mounted low-cost IMU, and zero-velocity constraint. Their indoor results could achieve an error of 1.2% of total distance travelled. In another work by Jimenez et al. [13], the reported positioning error, computed by mechanizing the inertial equations with zero-velocity constraint using a foot-mounted sensor, was about 1% after walking 360 m. For indoor applications, most of the efforts in improving the navigation solution are made on the heading estimate by using magnetic sensors [14,15,16], while Li et al. [17] tried to improve foot-mounted navigation by detecting turning motion from a sequence of footsteps. All of these authors used a single sensor package mounted on the foot. Nilsson et al. [18] presented a work that involved localization using two foot-mounted sensors. Works on waist-mounted PDR have been reported by Alvarez et al. [19] and Lan and Shih [20]. Meanwhile Diaz [21] discussed PDR algorithms based on pocket-mounted inertial sensors used to estimate step length from the leading leg pitch angle. Context-awareness detection also has been introduced to improve the navigation solution [22,23,24] by identifying the pedestrian’s activities such as walking, running, sitting, standstill, etc.

Inertial sensors are not only used in the field of navigation but also have been used in motion capture applications [25,26]. With the current size of MEMS inertial sensors, it is realizable to have sensors-embedded clothing. Sabatini [27] compared different sensor fusion and filtering techniques for tracking the orientations of human body parts using inertial/magnetic sensors. His results showed that the proposed filtering algorithm had similar performance to the commercial software. Ancillao et al. [28] reviewed different algorithms that use the inertial sensors to measure the ground reaction forces and suggested that artificial neural networks are more suitable than biomechanical modelling for prediction of ground reaction force. Ahmed and Tahir [29] proposed a modification of the Kalman filter for estimating the human body orientation using inertial sensors. These authors deweighted the sensor measurements from the axes that were corrupted by the linear acceleration and the reported results indicated a significant improvement in the orientation estimation. Recently, Lee et al. [30] reported pedestrian navigation using multiple inertial sensors distributed on human lower limbs and used the kinematics of the lower limbs to constrain the pedestrian motion. Their results indicated a positioning error of 1.25% of total distance travelled was reachable.

Most of the works [13,14,15,19,21,30] cited above utilized high-end commercial consumer grade MEMS sensors with costs in the order of thousands of US dollars [31], but it is not practical to equip pedestrians with expensive sensors. This type of sensor is designed to provide accurate inertial-based motion tracking [25] with orientation accuracy of 1.5 degrees RMS or less [32]. However, there exist inexpensive consumer grade (low-cost) inertial sensors with price tags of 20 US dollars or less when purchased in small quantities. This type of sensor is usually used by hobbyists for simple UAV navigation [33] and the orientation accuracy is definitely not as good as the high-end commercial sensor. They are also found in smartphones and game controllers. Shin et al. [34] attempted to use such low-cost MEMS sensors to develop an adaptive step length estimator. Their proposed algorithm used mathematical modelling to estimate the step length. Two examples of low-cost MEMS sensors are the MPU-6050 and MPU-9250 from TDK Invensense. Harle [9] mentioned that it is difficult to make a fair comparison between different positioning methods because of the lack of a unified set of testing parameters. Given the lack of research in pedestrian navigation using low-cost wearable sensors, this research attempts to investigate the performance of low-cost inertial sensors that are distributed on lower limbs.

The aim of this paper is to investigate the performance of low-cost sensors in estimating pedestrian step size and limb segment orientation as the inputs to the pedestrian dead-reckoning algorithm and compare this to existing PDR methods. This paper proposes a pedestrian navigation system that uses multiple low-cost inertial/magnetic sensors and the gait kinematics as an alternative to a single higher cost inertial sensor. The novelty of this work is the use of multiple low-cost sensors to model the kinematics of the lower limbs for computing the step sizes using the limb segment orientations. This paper presents the analysis and comparison of different step size estimations and the performance of the low-cost sensors in estimating the lower limb segment orientations.

The remainder of this paper is organized as follows: Section 2 introduces the developed wearable multi-sensor system, the human gait parameters, and the computational methods used in this paper. Section 3 describes the conducted experiments and results and discussion follow in Section 4 and Section 5.

## 2. Methodology

Based on the mechanization process, pedestrian navigation algorithms using self-contained sensors can be classified into two classes: Strapdown inertial navigation and step-heading systems [9,10]. The former method is based on traditional inertial navigation techniques which are ill-suited for low-cost sensors, while the latter method has the advantage of reducing the position error growth to a function of total travelled distance. The step-heading algorithms are generally divided into three components: Step detection, step size estimation, and position tracking. The last component involves fusing the subject’s displacement with the direction of the movement. In this work, the proposed methods use seven wearable sensors to measure the motions of lower limb segments. Forward kinematics of lower limbs are used to compute step sizes. This section discusses the developed wearable multi-sensor system, the related gait parameters, and, for comparison, the different dead-reckoning methods used in the pedestrian navigation literature.

### 2.1. Multi-Wearable Sensor System Overview

**Figure 1:**
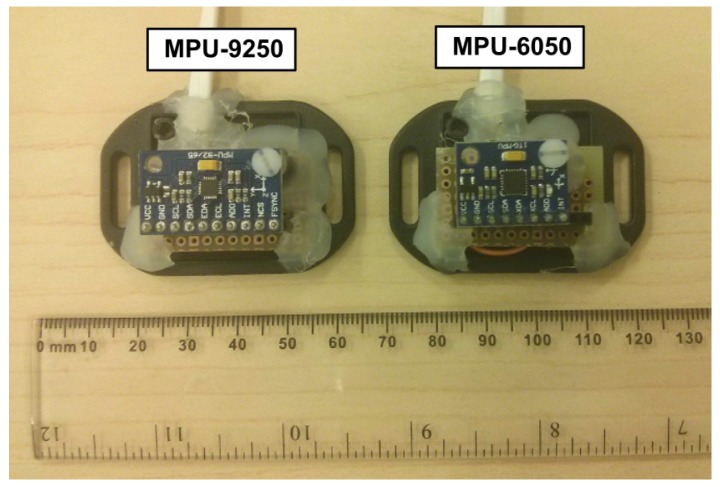
MPU-9250 and MPU-6050 sensor modules used in the experiment.

Inexpensive low-cost inertial/magnetic sensors often come in the form of breakout boards with integrated circuit chips. In order to use them, these modules must be wired and connected to a microcontroller. Figure 1 shows the two different sensors used in this research, MPU-6050 [35] and MPU-9250 [36] from TDK Invensense (San Jose, CA, USA). Both types of sensors have accelerometer and gyroscope triads, while only MPU-9250 has an additional magnetometer triad. The sensor specifications are listed in Table 1.

A data logging system capable of collecting raw measurements from multiple sensors was developed. The key components are a micro-controller, sensor boards, and a logging computer. A NUCLEO-F746ZG micro-controller from STMicroelectronics (Geneva, Switzerland) [37] was selected to read multiple sensor data. This device has four inter-integrated circuit (I2C) buses for sensor connections. Each of these buses can handle two sensor modules such that a pair of MPU-9250 and MPU-6050 is connected into one I2C bus. Two different sensors are paired together because an I2C bus cannot handle two magnetometers that have the same I2C address. In the sensor data logger developed for this paper, only the magnetometers mounted on the pelvis and right foot were used.

**Figure 2:**
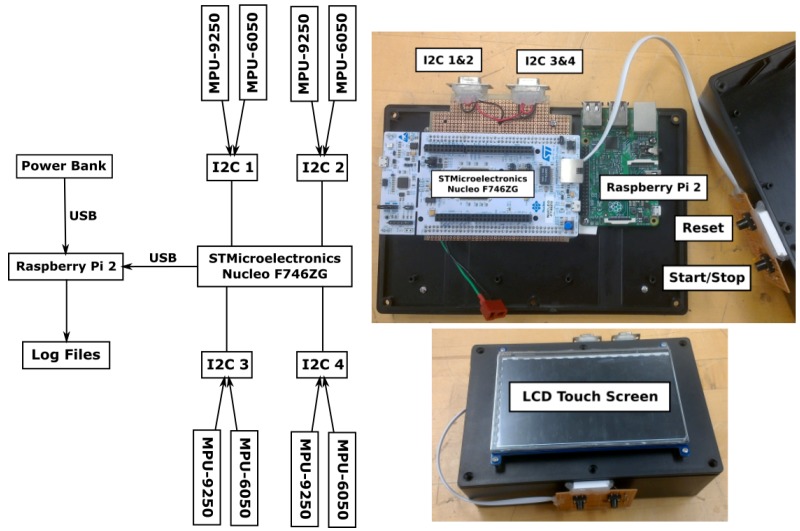
Sensor data logger system flowchart and its components.

Figure 2 shows the developed data logging system along with its program flow and components. This system is designed to be portable such that it can be easily carried by a test subject with less effect on the walking gait. There are eight sensor modules connected to one micro-controller using four I2C buses. All data are read in series and sent to a logging computer using serial port. The time stamps are from the micro-controller’s clock. A Raspberry Pi (Cambridge, UK) [38] is chosen for its compactness and is connected to a 7-inch LCD touch screen for user interfacing purposes. The entire system can be powered by a USB power bank. This data logging was capable of logging all eight sensors at a rate of approximately 26 Hz in this configuration.

### 2.2. Overview of Walking Gait

Before getting into the discussion of the proposed methods, it is important to introduce some parameters used in the gait kinematics that are related to the work in pedestrian navigation. For a more detailed description of gait, readers are advised to consult the book written by Vaughan et al. [39].

**Figure 3:**
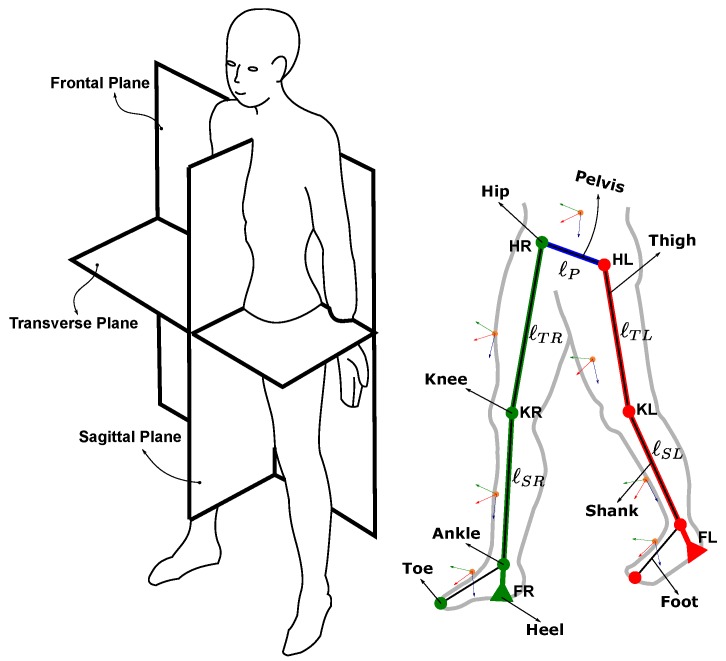
Human body coordinates and lower limb segments. (**left**) The human body can be divided into three orthogonal planes. (**right**) Skeletal model for lower limb segments and wearable sensor locations after Vaughan et al. [39].

Human gait kinematics describe the motion of the legs during locomotion. Figure 3 shows the human body coordinate system and the lower limb segments. The human body motion is usually described using a body-frame coordinate system consisting of three orthogonal planes: Frontal, sagittal, and transverse. The lower limbs can be divided into seven segments: Pelvis, a pair of thighs, shanks, and feet.

In the context of pedestrian navigation, the common gait parameters used are step length, stride length, and step frequency or cadence. From the literature, the step length and stride length along with the direction of walk are the inputs to the pedestrian dead-reckoning algorithm. In this paper, both step length and stride length are denoted as step size. The difference depends on how the step size is computed.

**Figure 4:**
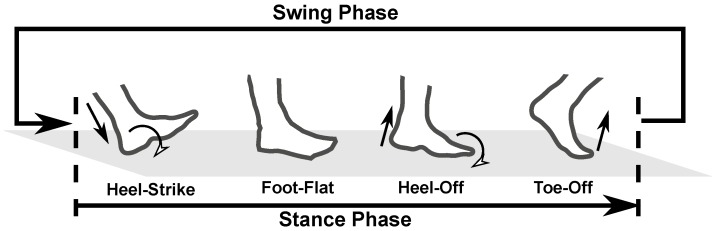
Detectable foot gait events and phases.

To compute the step size, a step detection is required to identify the swing and stance phases. The implemented foot gait event detection is able to detect different events during stance phase as shown in Figure 4. The swing phase is the duration between toe-off and heel-strike while the stance duration is between heel-strike and toe-off. The foot-flat events are the periods where the zero-velocity constraint is applied.

### 2.3. Navigation Methods

#### 2.3.1. Foot Gait and Quasi-Static Period Detections

In pedestrian dead-reckoning algorithms, a step detector is used to identify stance periods during walking. There are many step detection algorithms that have been developed for pedestrian navigation with the simplest detection being a step counter. In this paper, the methods developed by Sabatini et al. [40] and Skog et al. [41] will be used and compared. The former method uses the foot’s sagittal gyroscope signals and has the ability to detect different foot gait events: Heel-strike, foot-flat, heel-off, and toe-off, as mentioned in Section 2.2. Meanwhile, the latter method uses both accelerometer and gyroscope measurements from the foot and it is able to provide quasi-static period detection. A quasi-static period is an event where the foot is experiencing almost zero motion for a short period of time [42]. The quasi-static period is the same as the foot-flat period. By identifying the toe-off and heel-strike events, the lower limb motion can be segmented between stance and swing periods.

In this work, Skog’s generalized likelihood ratio test (GLRT) detector [41] is used to detect quasi-static periods based on both accelerometer and gyroscope signals. This algorithm detects the foot’s quasi-static periods based on the mean square error of the weighted sum of the acceleration and angular rate measurements. The detection is based on a chosen moving window size and the weighting is based on the chosen sensor noise values. The detection algorithm can be written as follows

(1) $$S_{k}=\frac{1}{N}\sum_{i=1}^{N}\left(\frac{1}{\sigma_{a}^{2}}{∥f_{i}-g\frac{{\bar{f}}_{i}}{∥{\bar{f}}_{i}∥}∥}^{2}+\frac{1}{\sigma_{\omega }^{2}}{∥\omega_{i}∥}^{2}\right)<T$$

where f and $\sigma_{a}$ are the accelerometer measurement vector and its noise standard deviation, ω and $\sigma_{\omega }$ are the gyroscope measurement vector and its noise standard deviation, $\bar{f}$ denotes the mean accelerometer measurement vector, and *N* is the window size. The quasi-static event is declared when the test value, $S_{k}$, is less then the threshold, *T*. This method can only detect foot-flat events and any irregular motions, which do not contribute significant position changes, can affect its performance. To improve the detector performance, minimum stance and swing periods are introduced such that the effect of irregular motion is minimized.

**Figure 5:**
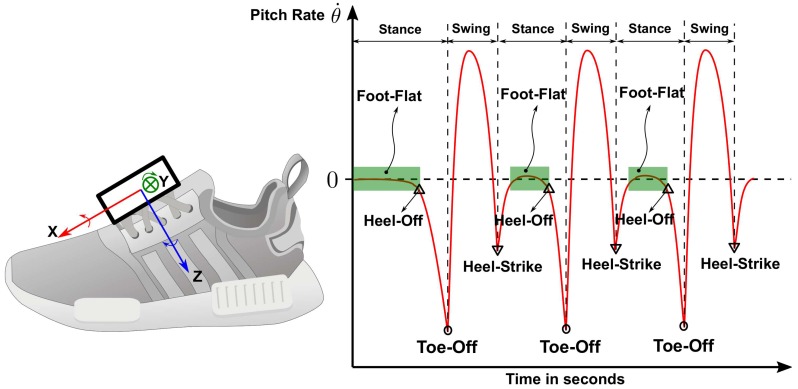
Foot-mounted sensor and its sagittal angular rate signal.

In the reference [43], the authors show that it is possible to estimate the step lengths by using the pitch angles of thighs and shanks during toe-off and heel-strike events. This requires the use of forward kinematics of lower limbs to compute the step sizes and an accurate gait segmentation is required, especially detecting toe-off and heel-strike. The method described by Equation (1) cannot detect these two gait events accurately because it performs gait segmentation based on foot-flat events. To detect toe-off and heel-strike, a method developed by Sabatini et al. [40] is adopted in this work. This foot gait segmentation assumes the subject is in normal walking mode and there is one foot sensor axis that is oriented and aligned with the body’s sagittal axis. As a result, the changes in the sagittal angular rate signals can be used to detect the gait cyclic events. The ideal normal walking sagittal angular rate signals of a foot-mounted sensor, in which its *x*-axis is oriented forward and *z*-axis is pointing downward, are drawn in Figure 5. The stance phase is marked by a period from heel-strike to toe-off, while the swing phase is from toe-off to heel-strike. Foot-flat events can be determined when the gyroscope values are around zero. The heel-strike and toe-off events are marked by the change of the slope of the angular rate signal from negative to positive. This detection method is able to detect four different foot gait events. However, this gait detection method often suffers from weak angular rate signals.

Gait segmentation and quasi-static detection methods implemented in this paper are summarized in Algorithm 1. Equation (1) is used to detect a complete gait cycle which consists of a pair of stance and swing phases. At each complete gait cycle, the gyroscope signals are being analyzed for the gait event detection. The gyroscope magnitude signal is used to mitigate weak rotation signals. A low-pass filter is used to remove any glitches that might affect the signal peak detection. During a normal/proper swing phase, the gyroscope magnitude signals usually contain three peaks, as shown in Figure 5. However, the toe-off and heel-strike peaks can be difficult to detect in the case of weak rotation and often only one peak is found. Hence, the condition of at least two peaks is selected to declare a proper swing phase in the detector implementation. The purpose of this gait detection is to have a better swing phase segmentation. A swing phase generates a stride length because this step size is defined as the distance between consecutive toe-off and heel-strike events of one particular foot. Therefore, the detections of foot-flat and heel-off using the gyroscope are optional and these are done for the completeness of the foot gait event identification.

**Algorithm 1** Foot gait event detection algorithm
1:**procedure**FootGaitEventDetector(time (t), accelerometer ($f_{ib}^{b}$), gyroscope ($\omega_{ib}^{b}$))2:    Define threshold for stance phase, *T* of Equation (1)3:    Define zero rotation threshold or tolerance value, $\omega_{0}$4:    Assume the initial and final poses are in *foot-flat* event and *stance* phase5:    Use Equation (1) to detect one gait cycle (a pair of *stance* and *swing* phases)6:    **for** each gait cycle **do**7:        Collect gyroscope data during this gait cycle $\to \omega_{ib,k}^{b}$8:        Compute gyroscope magnitude $\to ∥\omega_{ib,k}^{b}∥$9:        Apply 1^st^-order low-pass filter on the gyroscope magnitude $\to \omega_{k}$10:        Perform signal peak detection on $\omega_{k}$ during the swing period11:        **if** Number of peaks ≥ 2 **then**12:           Declare a good gait cycle is found or a step is taken13:           Mark the first peak as *toe-off*14:           Mark the last peak as *heel-strike*15:           Mark all signals satisfying $\omega_{k}<\omega_{0}$ from the start of stance phase as *foot-flat*16:           Mark the last signal satisfying $|\omega_{k}|\geq \omega_{0}$ from the end of stance phase as *heel-off*17:        **else**18:           Skip to the next gait cycle19:        **end if**20:    **end for**21:    Collect all time stamps in each foot gait event22:
**end procedure**



#### 2.3.2. Step Size Estimations Using Mathematical Models

A step length is defined as the distance between two feet when the leading foot strikes the ground during walking. Meanwhile, a stride length refers to the displacement of the swinging foot in one swing phase. One method to estimate step size is to fit mathematical models to the acceleration signals either from the pelvis or foot. These models usually have one tuning parameter that needs to be determined using a set of training data.

Weinberg [44] proposed step length estimation that is based on the vertical displacement of the hip during walking. In Weinberg’s model, the step length is modelled using the difference between the maximum and minimum acceleration values as follows

(2) $$\mathrm{Step}\;\mathrm{Length}=k\;\sqrt[{4}]{a_{\max}-a_{\min}}$$

where $a_{\max}$, $a_{\min}$, and *k* are the maximum and minimum acceleration values during one gait cycle, and a tuning parameter, respectively.

For the case of stride length, Kim et al. [45] proposed a mathematical model to estimate stride using a foot-mounted sensor. The authors claim that the stride lengths are related to the mean acceleration values during one stride cycle. This model is expressed as follows

(3) $$\mathrm{Stride}\;\mathrm{Length}=k\;\sqrt[{3}]{\frac{\sum_{i=1}^{N}\left|a_{i}\right|}{N}}$$

where *a* is the acceleration values during one gait cycle and *k* is a tuning parameter. In the reference [45], *k* is set to 0.98, but in this paper a new value of *k* will be determined using a set of training data.

Both acceleration values expressed in Equations (2) and (3) can be computed by subtracting the local gravitational acceleration value from the specific force magnitude.

(4) $$a_{i}=∥f_{ib,i}^{b}∥-g$$

#### 2.3.3. Simplified Strapdown Inertial Navigation

In strapdown inertial navigation, the inertial sensors record the motion through specific forces and angular rates. The details of inertial navigation computation can found in the popular navigation textbook [46]. In this paper, the low-cost inertial sensors are used and the inertial navigation equations can be rewritten as follows

(5) $$\left[\begin{array}{c} {\dot{C}}_{b}^{n}\\ {\dot{v}}_{eb}^{n}\\ {\dot{p}}_{eb}^{n} \end{array}\right]\approx \left[\begin{array}{c} C_{b}^{n}\left[\omega_{nb}^{b}\times \right]\\ C_{b}^{n}\;f_{ib}^{b}+g^{n}\\ v_{eb}^{n} \end{array}\right]$$

where the body rotational motion with respect to the local-level frame, $\omega_{nb}^{b}$, is assumed to be directly sensed by the raw gyroscope measurements, $\omega_{ib}^{b}$. In other words, the gyroscope measurements are assumed to be equivalent to the attitude rates. The symbol [•×] denotes the skew-symmetric matrix form of a vector. The subscripts and superscripts *n*, *b*, and *i* represent an arbitrary local-level frame (aligned in a forward–across–down), the sensor body frame, and the inertial frame, respectively.

#### 2.3.4. Limb Segment Orientation Estimation

Human lower limbs can be modelled using a skeletal model that is formed by seven segments connected by joints. Each of these segments can be mounted with one wearable sensor and these distributed sensors allow the rotational motions of lower limbs to be recorded. In this paper, the wearable sensors are used to estimate the orientation of each lower limb segment.

A step size can also be defined as how wide both legs are opened during walking. Knowing the sensor orientations, the lower limb pitch angles can be estimated and the step size can also be computed based on the sensor pitch angles provided the lengths of the segments are known.

In this work, a Kalman filter framework is used to integrate the angular rate measurements from the lower limb segments and the to determine sensor orientation angles. These orientations can be described by a set of three attitude angles following the so-called “aerospace sequence” [47] formed by roll, pitch, and yaw angles. This filter is designed to track the orientations of five limb segments: The pelvis, the left and right thighs, and the left and right shanks. The orientation of the foot or ankle does not give much information for step size computation. The state vector consists of three parts: Attitude angles, attitude rates, and angular rate biases for each of the five segments.

(6) $$x={\left[\begin{array}{ccc} \Psi_{nb} & \omega_{nb}^{b} & b_{\omega }^{b} \end{array}\right]}^{T}$$

where each term of Equation (6) is defined as follows

$$\begin{array}{cc} \Psi_{nb} & ={\left[\begin{array}{ccccc} \Psi_{nb,P} & \Psi_{nb,\mathrm{TL}} & \Psi_{nb,\mathrm{TR}} & \Psi_{nb,\mathrm{SL}} & \Psi_{nb,\mathrm{SR}} \end{array}\right]}^{T}\\ \omega_{nb}^{b} & ={\left[\begin{array}{ccccc} \omega_{nb,P}^{b} & \omega_{nb,\mathrm{TL}}^{b} & \omega_{nb,\mathrm{TR}}^{b} & \omega_{nb,\mathrm{SL}}^{b} & \omega_{nb,\mathrm{SR}}^{b} \end{array}\right]}^{T}\\ b_{\omega }^{b} & ={\left[\begin{array}{ccccc} b_{\omega ,P}^{b} & b_{\omega ,\mathrm{TL}}^{b} & b_{\omega ,\mathrm{TR}}^{b} & b_{\omega ,\mathrm{SL}}^{b} & b_{\omega ,\mathrm{SR}}^{b} \end{array}\right]}^{T} \end{array}$$

The subscripts P, T, and S refer to pelvis, thigh, and shank, respectively. The left and right legs are represented by subscript L and R, respectively. Each attitude vector, $\Psi_{nb,•}$, consists of three orientation angles: Roll (ϕ), pitch (θ), and yaw (ψ). Each attitude rate vector ($\omega_{nb,•}^{b}$) and gyroscope bias vector ($b_{\omega ,•}^{b}$) has a size of 3×1, representing the three orthogonal axes. In this method, it is worth mentioning that the attitude rate vector is assumed to be equivalent to the gyroscope measurement due to the inertial sensor used, $\omega_{nb,•}^{b}\approx \omega_{ib,•}^{b}$.

The filter prediction is based on a linear system dynamics model and its state-space representation can be written as follows

(7) $$\underset{{\hat{x}}_{k+1}^{-}}{\underset{︸}{{\left[\begin{array}{c} \Psi_{nb}\\ \omega_{nb}^{b}\\ b_{\omega }^{b} \end{array}\right]}_{k+1}}}=\underset{\Phi_{k,k+1}}{\underset{︸}{\left[\begin{array}{ccc} I_{15} & \Delta t\;I_{15} & 0_{15\times 15}\\ 0_{15\times 15} & I_{15} & 0_{15\times 15}\\ 0_{15\times 15} & 0_{15\times 15} & I_{15}-\beta_{\omega }\Delta t \end{array}\right]}}\;\underset{{\hat{x}}_{k}^{+}}{\underset{︸}{{\left[\begin{array}{c} \Psi_{nb}\\ \omega_{nb}^{b}\\ b_{\omega }^{b} \end{array}\right]}_{k}}}+\;Q_{k}$$

where $Q_{k}$ represents the process noise matrix and $\beta_{\omega }$ is a diagonal matrix that contains the inverse of correlation time of each sensor. The system modelling error is affected by the gyroscope noise and its bias drift. The gyroscope noise is modelled as random walk while the bias state is modelled as first order Gauss-Markov process.

(8) $$Q_{k}=\left[\begin{array}{ccc} \frac{1}{3}\Delta t^{3}\;S_{\omega } & \frac{1}{2}\Delta t^{2}\;S_{\omega } & 0_{15\times 15}\\ \frac{1}{2}\Delta t^{2}\;S_{\omega } & \Delta t^{2}\;S_{\omega } & 0_{15\times 15}\\ 0_{15\times 15} & 0_{15\times 15} & \Delta t\;S_{b_{\omega }}-\Delta t^{2}\;S_{b_{\omega }}\;\beta_{\omega }+\frac{1}{3}\Delta t^{3}\;S_{b_{\omega }}\;\beta_{\omega }^{2} \end{array}\right]$$

where $S_{\omega }$ and $S_{b_{\omega }}$ are diagonal matrices of power spectral densities of gyroscope noises and biases from each sensor, respectively.

In this orientation filter, the measurement updates are obtained from the available sensor measurements.

$$\begin{array}{cc} f_{ib}^{b} & ={\left[\begin{array}{ccc} f_{x} & f_{y} & f_{z} \end{array}\right]}^{T}\\ {\tilde{\omega }}_{ib}^{b} & ={\left[\begin{array}{ccc} \omega_{x} & \omega_{y} & \omega_{z} \end{array}\right]}^{T}\\ m^{b} & ={\left[\begin{array}{ccc} m_{x} & m_{y} & m_{z} \end{array}\right]}^{T} \end{array}$$

The accelerometer’s specific force ($f_{ib}^{b}$) is used to compute roll and pitch angles. The gyroscope measurements (${\tilde{\omega }}_{ib}^{b}$) are used to update the attitude rate state vector. In this paper, the heading angle is updated using a magnetic field measurement ($m^{b}$) when it is available and it is simply used to constrain the drift in the yaw estimate as this state is not otherwise observable in the filter. The measurement update equations are

(9) $$\phi =\arctan\left(\frac{-f_{y}}{-f_{z}}\right)+v_{\phi ,•}$$

(10) $$\theta =\arctan\left(\frac{f_{x}}{\sqrt{f_{y}^{2}+f_{z}^{2}}}\right)+v_{\theta ,•}$$

(11) $$\begin{array}{cc} \psi & =\arctan\left(\frac{-m_{y}\cos\phi +m_{z}\sin\phi }{m_{x}\cos\theta +m_{y}\sin\phi \sin\theta +m_{z}\cos\phi \sin\theta }\right)+v_{\psi ,•} \end{array}$$

(12) $${\tilde{\omega }}_{ib,•}^{b}=\omega_{ib,•}^{b}+b_{\omega ,•}^{b}+v_{\omega ,•}$$

where the parameter $v_{•}$ represents the measurement uncertainty. The rest of these filter equations follow the standard Kalman filter equations.

#### 2.3.5. Forward Kinematics of Lower Limbs

In gait analysis, the kinematics of the human body can be modelled as solid links connected with each other through joints. These links represent the body segments and their mobilities. Other aspects of motion analysis that are related to rigid body inertia are neglected. This type of kinematic model is classified as “anthropomorphic” or “skeletal” [48]. In navigation applications, only the lower limb segments need to be modelled and this skeletal model consists of five body segments: Pelvis and thighs and shanks from the left and right legs. The foot segments are neglected and replaced by an extended shank segment starting from the knee to the heel. This is done to simplify the forward kinematics because the foot does not contribute large position displacement in the step size as long as the heel position is considered in the computation.

A 2D step length estimation using lower limb pitch angles has been evaluated in the authors’ previous work [43]. The results indicated that the trailing shank pitch angle plays a significant role for getting accurate step length. The step size error on the sagittal plane when ignoring the trailing shank pitch is proportional to

(13) $$\delta {\mathrm{Step}}_{\mathrm{sagittal}}=\ell_{S}\;(\sin|\theta_{S}\left|-\sin\right|\theta_{T}\left|\right)$$

where $\theta_{T}$ and $\theta_{S}$ refer to the trailing limb’s thigh and shank pitch angles. Comparing the pitch angles of trailing thigh and shank, the ratio of these two angles around toe-off events is about two to one. Therefore, it is possible to model the trailing shank pitch angle by using the pitch angle of the thigh alone. The step length on the sagittal plane using the leading thigh’s pitch angle only can be modelled as

(14) $${\mathrm{Step}\;\mathrm{Length}}_{\mathrm{sagittal}}=\left(\ell_{T}+\ell_{S}\right)\sin|\theta_{T}|+\ell_{T}\sin|\theta_{T}|+\ell_{S}\sin\left|2\;\theta_{T}\right|$$

The skeletal model of lower limbs is shown in Figure 3. This model consists of four joints: Two hips and two knees. The hip joints are connected by the pelvis and this segment is allowed to perform rotation with three degrees of freedom. The hip and knee joints are modelled as single axis revolute joints which can only rotate in the sagittal plane to simplify the model of forward kinematics. The computation of forward kinematics requires one end of the links to be fixed while the other end is set free to move. This condition fits the cyclic nature of the walking cycle (stance and swing). The forward kinematics can be applied by setting the fixed end to be the foot in the stance phase, while the other end is the swinging foot during walking. This setup alternates as the swing and stance alternate during walking.

**Figure 6:**
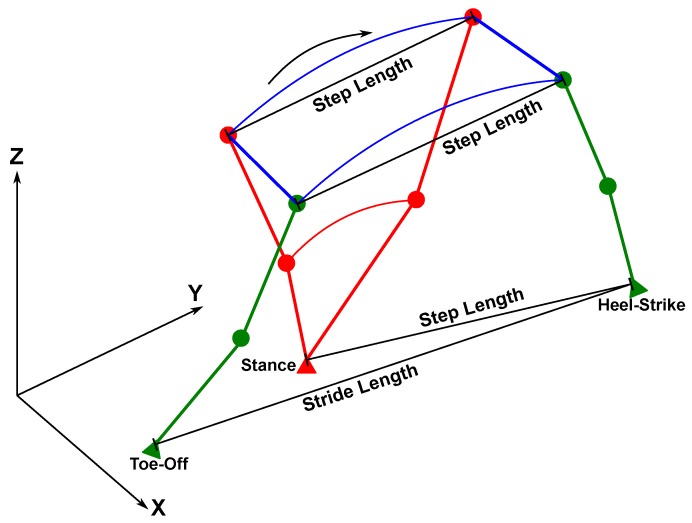
Step and stride lengths extracted from a solution set of forward kinematics. In this case, the left foot is in stance phase while the right foot is in the swing phase.

Given the lengths and orientations of each segment, the computation of forward kinematics starts from the fixed foot and follows the skeletal linkages until the other foot. According to the skeletal model, this computation requires all attitude angles of the pelvis and the pitch angles of the thighs and shanks. Assuming the right foot is in stance phase and the left foot is in swing phase, the forward kinematics equations can be written as follows

(15) $$\begin{array}{cc} p_{FR}^{n} & ={\left[\begin{array}{ccc} 0 & 0 & 0 \end{array}\right]}^{T} \end{array}$$

(16) $$\begin{array}{cc} p_{KR/FR}^{n} & =C_{b,SR}^{n}\;\ell_{SR}^{b} \end{array}$$

(17) $$\begin{array}{cc} p_{HR/FR}^{n} & =p_{KR/FR}^{n}+C_{b,TR}^{n}\;\ell_{TR}^{b}=C_{b,SR}^{n}\;\ell_{SR}^{b}+C_{b,TR}^{n}\;\ell_{TR}^{b} \end{array}$$

(18) $$\begin{array}{cc} p_{HL/FR}^{n} & =p_{HR/FR}^{n}+C_{b,P}^{n}\;\ell_{P}^{b}=C_{b,SR}^{n}\;\ell_{SR}^{b}+C_{b,TR}^{n}\;\ell_{TR}^{b}+C_{b,P}^{n}\;\ell_{P}^{b} \end{array}$$

(19) $$\begin{array}{cc} p_{KL/FR}^{n} & =p_{HL/FR}^{n}+C_{b,TL}^{n}\;\ell_{TL}^{b}=C_{b,SR}^{n}\;\ell_{SR}^{b}+C_{b,TR}^{n}\;\ell_{TR}^{b}+C_{b,P}^{n}\;\ell_{P}^{b}+C_{b,TL}^{n}\;\ell_{TL}^{b} \end{array}$$

(20) $$\begin{array}{cc} p_{FL/FR}^{n} & =p_{KL/FR}^{n}+C_{b,SL}^{n}\;\ell_{SL}^{b}=C_{b,SR}^{n}\;\ell_{SR}^{b}+C_{b,TR}^{n}\;\ell_{TR}^{b}+C_{b,P}^{n}\;\ell_{P}^{b}+C_{b,TL}^{n}\;\ell_{TL}^{b}+C_{b,SL}^{n}\;\ell_{SL}^{b} \end{array}$$

where the subscripts *F*, *K*, and *H* represent the foot, knee, and hip nodes in the skeletal model, while the subscripts *L* and *R* refer to the left and right sides. The segments are denoted by *P*, *T*, and *S* for pelvis, thigh, and shank, respectively. The position vectors are expressed in the local-level frame, denoted by $p_{•}^{n}$, and the symbol $C_{b,•}^{n}$ denotes the direction cosine matrix transforming from each sensor body frame to the local-level frame. Figure 6 shows the local-level frame. The limb segment vectors are expressed in the sensor body frame and written as $\ell_{•}^{b}$. The direction cosine matrix transformation from the sensor body frame to the local-level frame can be expressed as follows

(21) $$C_{b,•}^{n}={\left(\begin{array}{ccc} \cos\theta \cos\psi & -\cos\phi \sin\psi +\sin\phi \sin\theta \cos\psi & \sin\phi \sin\psi +\cos\phi \sin\theta \cos\psi \\ \cos\theta \sin\psi & \cos\phi \cos\psi +\sin\phi \sin\theta \sin\psi & -\sin\phi \cos\psi +\cos\phi \sin\theta \sin\psi \\ -\sin\theta & \sin\phi \cos\theta & \cos\phi \cos\theta \end{array}\right)}_{•}$$

where ϕ, θ, and ψ are the attitude angles estimated using the low-cost sensors (Section 2.3.4). For the case where the left foot is in stance phase and the right foot is in swing phase, the forward kinematics equations need to be adjusted by swapping the subscripts *L* and *R*.

The step sizes can be extracted from the solutions of the forward kinematics between the toe-off and heel-strike events, which bound the swing phases. A stride length is the foot position displacement from toe-off to heel-strike. A step length can be extracted in two different ways: The position displacement of the hip during stance phase or the swing phase, and the distance between the two feet when the swinging foot hits the ground (heel-strike) as shown by Equation (20). Figure 6 sketches the poses of the lower limbs during toe-off and heel-strike.

#### 2.3.6. Foot Strapdown Inertial Navigation Filter

One common method used in foot-mounted inertial navigation is to track the relative position of the foot. This can be done through strapdown inertial navigation. The advantages of the foot-mounted system are twofold. First, the foot gait information can be easily detected using the signal patterns of accelerometer and gyroscope triads. Second, the quasi-static periods during stance phases can be easily detected and these periods are used to apply the zero measurement updates in the navigation filter. This strapdown algorithm is presented in this paper for the completeness of comparison between different pedestrian navigation methods.

The system model of the filter is based on error perturbation of a constant velocity model. Due to the nonlinear system model, an extended Kalman filter is used. This filter is designed to estimate the error state vector and these outputs are used to correct the navigation state vector. The states of interest are the foot’s attitude angles, velocity, and position in the navigation frame. These parameters can be expressed as

(22) $$x={\left[\begin{array}{ccc} \Psi_{nb} & v_{eb}^{n} & p_{eb}^{n} \end{array}\right]}^{T}$$

Meanwhile the error state vector estimated by the Kalman filter is

(23) $$\delta x={\left[\begin{array}{ccc} \delta \Psi_{nb} & \delta v_{eb}^{n} & \delta p_{eb}^{n} \end{array}\right]}^{T}$$

In this implementation, the navigation filter does not estimate the sensor bias states because this filter is mainly used to estimate the stride length produced by the foot’s swing. This means that the filter will be reset for each stride and there will be only one zero-velocity update at the end of each swing.

Based on Equations (5) and (23), the state transition matrix can be expressed as

(24) $$\Phi_{k-1,\;k}=\left[\begin{array}{ccc} I_{3} & 0_{3\times 3} & 0_{3\times 3}\\ -\left[f_{ib}^{n}\times \right]\;\Delta t & I_{3} & 0_{3\times 3}\\ 0_{3\times 3} & \Delta t\;I_{3} & I_{3} \end{array}\right]$$

where $\left[f_{ib}^{n}\times \right]$ is the skew-symmetric matrix formed by specific force vector. In modelling the system error, the attitude angles and velocity vector are affected by gyroscope and accelerometer random walk errors. The system process noise matrix can be computed as follows

(25) $$Q_{k-1,\;k}=\int_{t_{k-1}}^{t_{k}}\Phi_{k-1,\;k}\left[\begin{array}{ccc} S_{rg} & 0_{3\times 3} & 0_{3\times 3}\\ 0_{3\times 3} & S_{ra} & 0_{3\times 3}\\ 0_{3\times 3} & 0_{3\times 3} & 0_{3\times 3} \end{array}\right]\Phi_{k-1,\;k}^{T}\;d\tau$$

where $S_{rg}$ and $S_{ra}$ represent the diagonal matrices of power spectral densities of gyroscope and accelerometer noises, respectively.

The Kalman filter measurement update comes from the zero-velocity pseudo-measurement. The measurement update and its design matrix can be expressed as

(26) $$\delta z_{k}=0_{3\times 1}-v_{eb}^{n-}$$

(27) $$H_{k}=\left[\begin{array}{ccc} 0_{3\times 3} & -I_{3} & 0_{3\times 3} \end{array}\right]$$

where $v_{eb}^{n-}$ refers to the velocity estimated from Equation (5). The optimal estimation algorithm follows the standard extended Kalman filter equations for inertial navigation. The foot position trajectory is smoothed using a common optimal smoothing method, Rauch-Tung-Striebel (RTS) smoother [49].

## 3. Experiments

**Figure 7:**
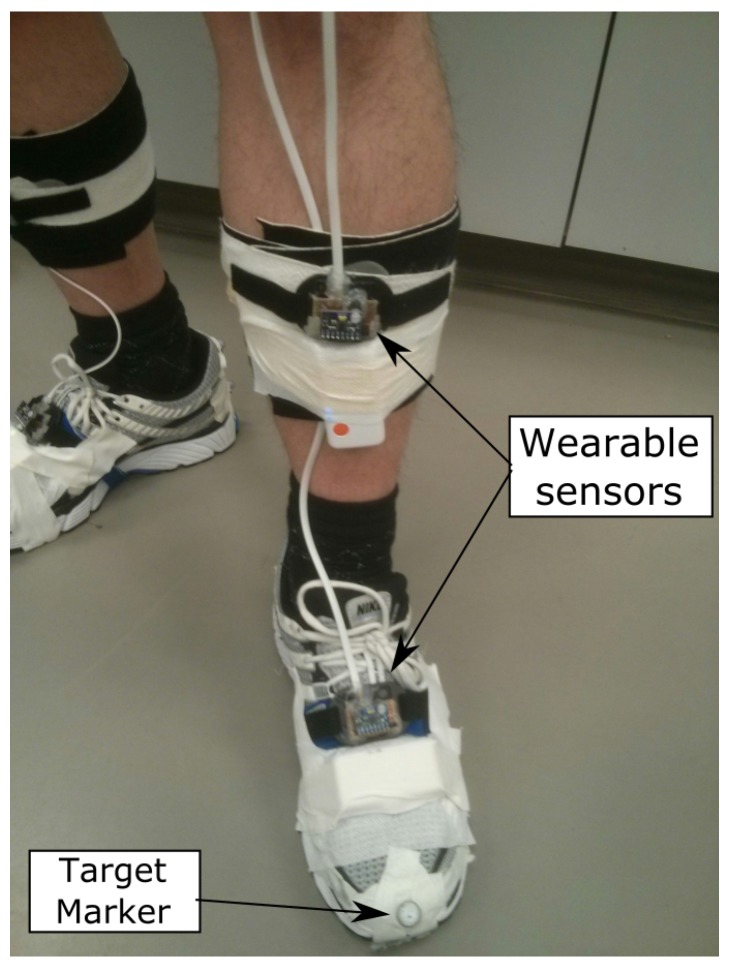
Wearable sensors mounted on the shank and foot with a Vicon marker for optical motion capture system [43].

The wearable multi-sensor system and the proposed methods were evaluated using a treadmill equipped with an optical motion capture system. This motion capture system consists of eight high-speed digital video cameras (Vicon MX3/Nexus, Vicon, Oxford, UK) and was used to record the positions of spherical retro-reflective markers (9 mm diameter, Mocap Solutions, Huntington Beach, CA, USA) that were distributed on the lower limbs. The setup of the optical motion capture system was similar to the setup described by Pohl et al. [50] and Watari et al. [51]. The sampling rate was set to 200 Hz. The results of this optical system are used to evaluate the results computed from the wearable sensors. Figure 7 shows how the wearable sensors are mounted on the shank and foot as well as a Vicon marker located on the toe. Three datasets, each approximately 90 s in duration, were collected.

The optical motion capture system reported the position histories of the markers, the orientation of the pelvis, and the joint angles of the hips and knees. The reference value of the step length is computed using the difference between the two heels’ locations at the moment of heel-strike. Meanwhile, the reference value of the stride length is computed as the distance between the position of the leading heel at heel-strike and its prior position at toe-off. The reference pelvis orientation is directly related to the pelvis joint angles. The reference pitch angles of thighs and shanks are constructed using the joint angles from the hips and knees.

The sensor placement locations are shown in Figure 3 as forward–across–down coordinate axis triads located on the back of the pelvis and on the front of each thigh, shank, and foot. A total of seven wearable sensors is used. The right leg segments and pelvis are mounted with MPU-9250s, while the left leg segments are mounted with MPU-6050s. All sensors are tightly strapped to the limb segments to minimize the effect of tissue motion during walking.

The sensor outputs are used in orientation estimation, described in Section 2.3.4, where the results are represented by the attitude angles. These angles are used in Equation (21) to transform the positions of the feet, knees, and hips from the sensor body frames to the local-level frame. This estimated local-level frame differs from the reference Vicon local-level frame by a translation and an arbitrary rotation about the vertical.

## 4. Results

### 4.1. Foot Gait Segmentation and Quasi-Static Detection

The performances of the detectors described by Equation (1) and Algorithm 1 are summarized in Table 2 in terms of the number of swing phases detected. The reference number of swings is based on the number of times that the foot leaves the ground as detailed in the optical motion capture data. In general, both detectors perform very well in identifying stance and swing periods with an error of less than 3%.

**Figure 8:**
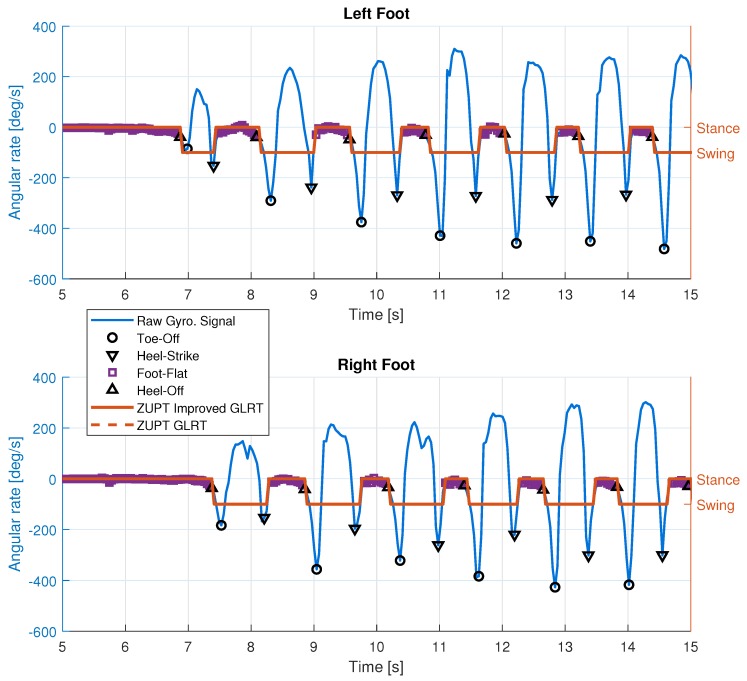
Results of foot gait events and quasi-static periods for left foot (**top**) and right foot (**bottom**). The gyroscope measurements shown are the signals on the sagittal axis.

**Figure 9:**
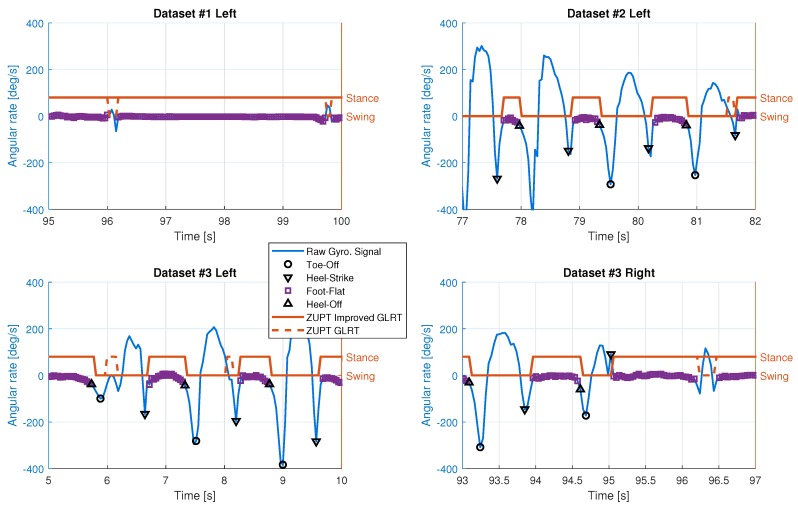
Missed and false step detections from three datasets. (**top left**) False swing detections from left foot of Dataset #1. (**top right**) False stance detection from right foot of Dataset #2. (**bottom left**) False stance detections from left foot of Dataset #3. (**bottom right**) Missed swing detection from right foot of Dataset #3.

Figure 8 shows the results of foot gait identification and quasi-static period detection from Dataset #2. This figure clearly shows the cyclic nature of the human walking gait and the alternations between the left and right legs during walking. The missed and false detections are caused by the weak rotation signals that occasionally occur during the beginning and the end of walking periods. Figure 9 shows a visual description of the missed and false detections. The detection results from Equation (1) are very sensitive to the outputs of both accelerometer and gyroscope, as shown in the top left plot of Figure 9. The bottom right plot shows the effect of weak right foot motion to the Algorithm 1 detector.

The results of foot quasi-static periods were used to determine periods when virtual measurements (zero-velocity and angular rate updates) are applied in the Kalman filter. The results of foot gait event identification are used to determine when swing phases happen. This is important because a stride length is defined as the foot position displacement caused by the swing of the particular leg and it is marked by a period from toe-off to heel-strike. In order to use forward kinematics of lower limbs for computing stride lengths, the time stamps of toe-off and heel-strike must be determined accurately from the gait segmentation.

### 4.2. Lower Limb Segment Orientations

**Figure 10:**
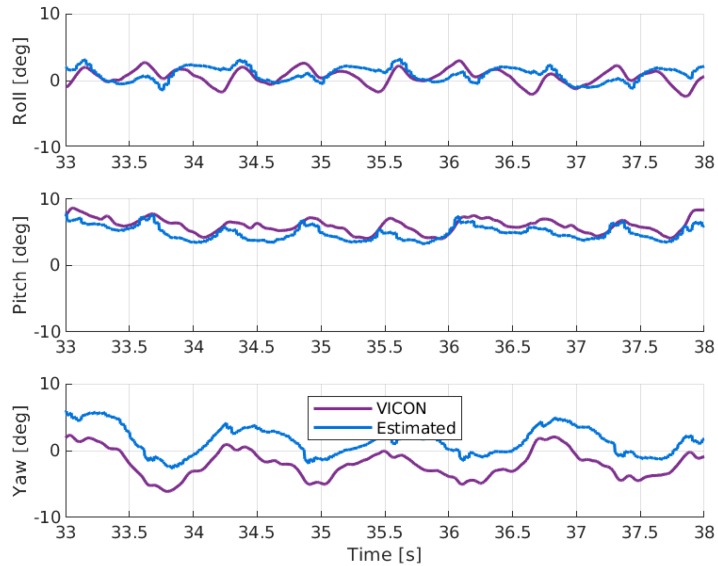
Comparison of roll, pitch, and yaw angles on pelvis segment during walking.

**Figure 11:**
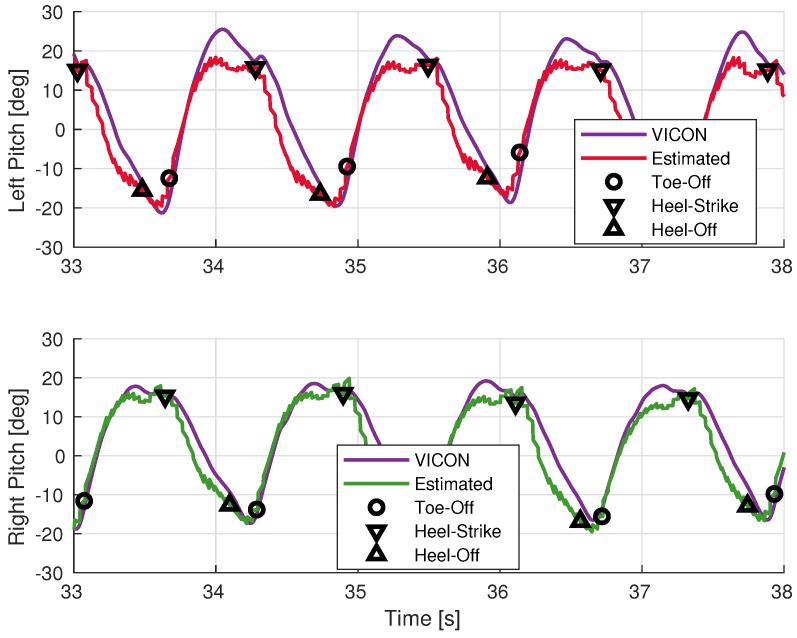
Comparison of thigh pitch angles during walking.

**Figure 12:**
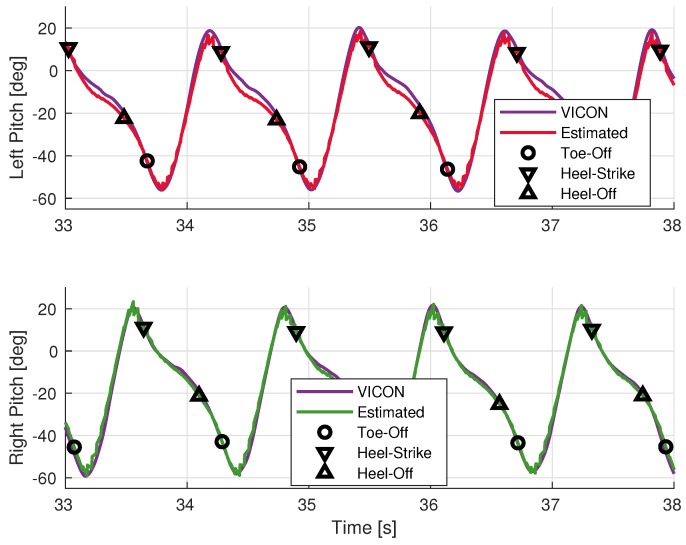
Comparison of shank pitch angles during walking.

The results of the sensor orientation filter described in Section 2.3.4 are shown in Figure 10, Figure 11 and Figure 12. The error statistics are summarized in Table 3. The overall estimated segment orientation has a mean error of less than 6 degrees.

The pelvis orientations (Figure 10) show that the filter is not able to track the rolling motion of the pelvis segment. The Kalman filter is designed to do integration on the attitude rates in order to get orientation angles. During walking, the pelvis rotational motion on frontal plane is very small and the low-cost inertial sensor may not be able to detect it. Looking at the magnitude of the rolling motion, one possible explanation is that the gyroscope noise is larger than the true rotation signal such that the filter mainly integrates the sensor noise. For the other two planes, the pelvis pitch and yaw angles are able to follow the general trends of the reference data.

The thigh and shank pitch angles can be estimated well with these low-cost inertial sensors, as shown in Figure 11 and Figure 12. The leading limb segments have positive pitch angles while the trailing segments have negative values. The heel-strike events are located around the maximum pitch, while the toe-off events are around the minimum pitch.The leading leg is almost straight during the heel-strike shown by the pitch angles of the thigh and shank being almost the same. However, this is not the case for the trailing leg, especially for the shank. This confirms, prior to observation, that estimating step sizes using thigh-mounted sensors must take into account the effect of the pitch angle of the trailing shank.

According to the results, the human walking motion is mainly captured on the sagittal plane. The rotational motions on the frontal and transverse planes are very weak, except the pelvis, which rotates mostly on the sagittal and transverse planes. The roll and yaw angles of the thighs and shanks are difficult to estimate due to their weak rotation signals and the quality of the inertial sensors used. Furthermore, the yaw angles estimation will depend on the existence of a magnetometer triad because this state is not observable in an inertial-based filter using only MEMS IMUs.

There are offsets between the estimated attitude angles and the reference data from the optical system. These offsets are caused by imperfections in the sensor error model and are due to the limitation where it is impossible to put the sensors on the centres of the gravity of each limb segment. These types of errors can be reduced by performing sensor-to-body coordinate alignment [52]. The human tissue also plays a role in the pitch angle estimation, especially on the thigh segments. The volume of the thigh segment is much larger than the shank and this causes the effect of tissue movement on the thigh to degrade the thigh pitch angle estimation compared to the shank pitch angle.

### 4.3. Forward Kinematics Results

**Figure 13:**
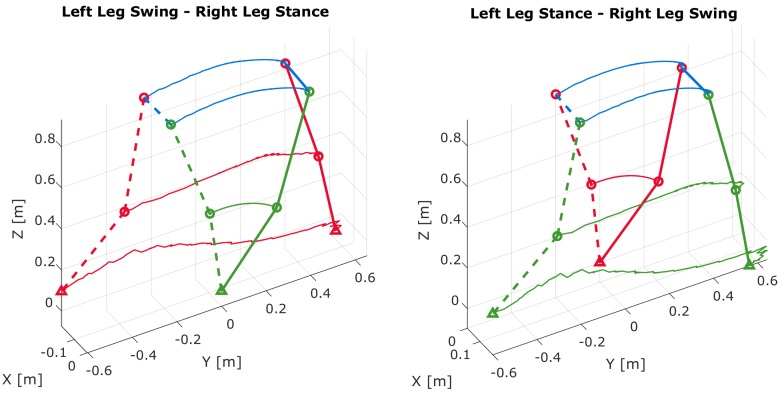
Forward kinematics of skeletal model between toe-off and heel-strike: (**left**) Left limb swing and right limb stance. (**right**) Right limb swing and left limb stance. Dashed lines refer to the start of the swing phase (toe-off) and solid lines refer to the end of swing phase (heel-strike).

Forward kinematics allow the estimation of positions of joints given a skeletal model. Figure 13 shows how walking progresses during stance and swing phases. These results are generated by evaluating the positions of the joints using Equations (15)–(20). In the human walking gait cycle, there is always one foot in stance phase while the other foot is in swing phase. This figure clearly shows that the trailing shank pitch angle does have a significant effect on the step size computation.

**Figure 14:**
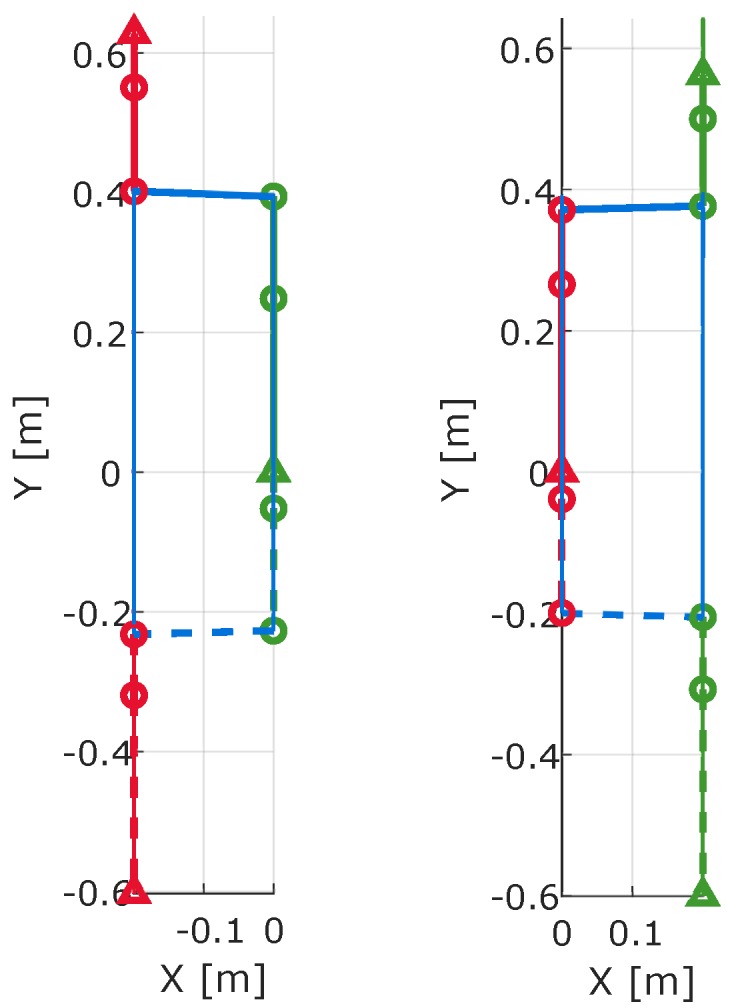
Top view of forward kinematics of skeletal model between toe-off and heel-strike: (**left**) Left limb swing and right limb stance. (**right**) Right limb swing and left limb stance.

Figure 14 provides a top view of Figure 13. There exist small differences in hip-based step lengths between the left and right limbs. The hip step length computed from the stance limb is less than the one computed from the swing limb. These differences are due to the pelvis yaw angles during toe-off and heel-strike.

Since the trajectories of joints are traceable, the forward kinematics not only provide a position update but also give heading information during one stride. However, the discussion about using this heading information will be left for future work because the current experiment does not include any heading changes. Furthermore, it is also possible to enhance the detection and estimation of pedestrian turns using the forward kinematics by tracking the foot position.

### 4.4. Comparison of Estimated Step Sizes

The tuning parameters, *k*, from the mathematical model-based methods are determined using Dataset #2. The first and the last steps of all datasets are excluded in the error computation due to the acceleration and deceleration of the treadmill and the fact that the gait patterns during these periods are usually not consistent with the rest of the data. The mathematical models of step sizes are based on the acceleration values and the walking speed affects these acceleration signals directly. Faster walking speed tends to have higher acceleration. Thus, the parameters, *k*, depend on the step frequency as well.

**Figure 15:**
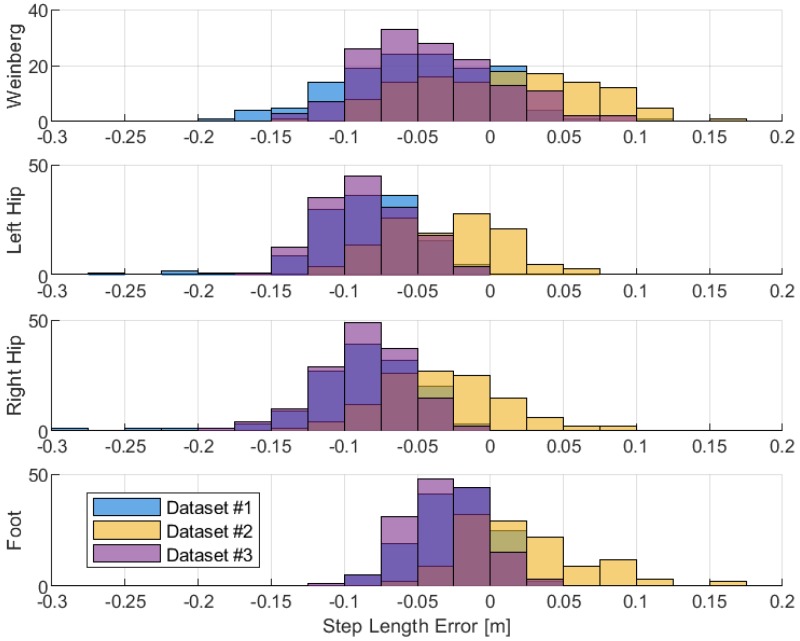
Step length error distributions between different methods.

The different step length estimations are compared in Figure 15 and Table 4. The estimated step length using the mathematical model suffers from the incorrect model represented by the value of *k*. It is difficult to estimate step length accurately using the mathematical model because each person has different gait patterns. The implementation of this mathematical model usually requires constant tuning of the model, for which a good GNSS positioning solution is needed. The step length estimated using 3D forward kinematics has smaller mean error compared to other methods. A mean error of 3.2 cm is achievable by using low-cost inertial sensors. Here, the footstep length is treated as a three-dimensional distance between the left and right heels. The step length errors estimated from the hip position are slightly larger because the reference step length is computed based on the position of heels recorded by the optical motion capture system.

**Figure 16:**
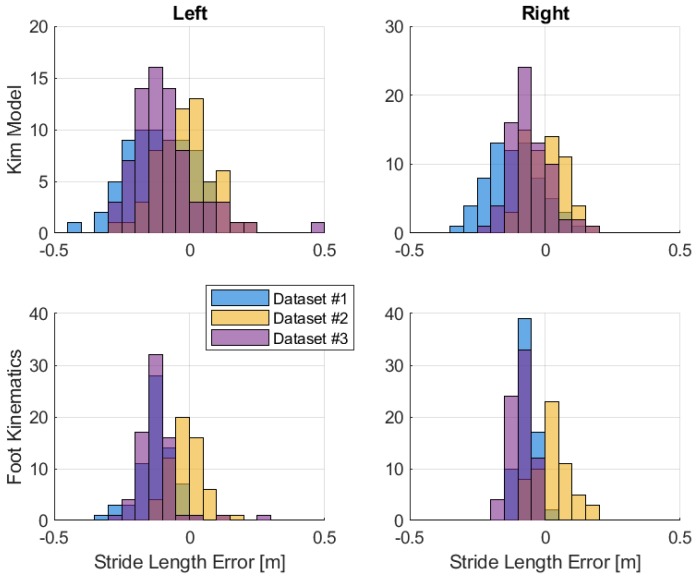
Stride length error distributions between different methods.

The stride length estimations are compared in Figure 16 and Table 5. The results of stride length computed using forward kinematics have less variations compared to the mathematical model. However, the mean stride length errors are similar. The method of forward kinematics does not depend on model tuning, but it does rely on the segment orientation estimation and knowledge of segment length. Looking at the results of forward kinematics, the left stride tends to have larger error than the right. The differing sensor models may cause this effect.

The performance of the pedestrian navigation algorithm is usually expressed in terms of percentage of the total travelled distance. Since the walking experiment is conducted on the treadmill, the total travelled distances are computed using the sum of the step lengths from the optical motion capture system and the developed multi-sensor system. The positioning errors of the three datasets are 3.7% of 89.156 m, 3.6% of 72.795 m, and 4.9% of 94.761 m, respectively.

### 4.5. Foot Strapdown Inertial Algorithm Using Low-Cost Sensors

**Figure 17:**
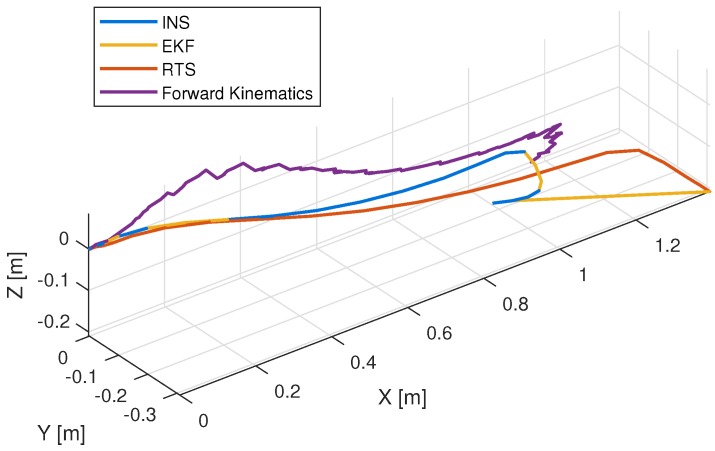
Right foot position trajectory during a swing.

The results of foot strapdown algorithm using low-cost sensors are expected to be error-prone. This is due to the fact that these low-cost sensors have larger noise levels than the more expensive sensors usually used with strapdown algorithms. Performing double integration on noisy acceleration signals can lead to large position error. Figure 17 shows the foot position trajectories during a swing phase where the result of the strapdown algorithm has drifted about 30 cm to the right. This drift can lead to navigation error in position and azimuth. A Rauch–Tung–Striebel (RTS) smoother is also used to generate smoothed foot trajectory, but it does not change the estimated stride length. On the other hand, the foot position computed using forward kinematics that is based on sensor orientations does not drift in the horizontal plane, but does drift in the vertical axis. This drift is due to the error caused by the simplification of the skeletal model in which the motion of the foot segment is ignored in the computation.

**Figure 18:**
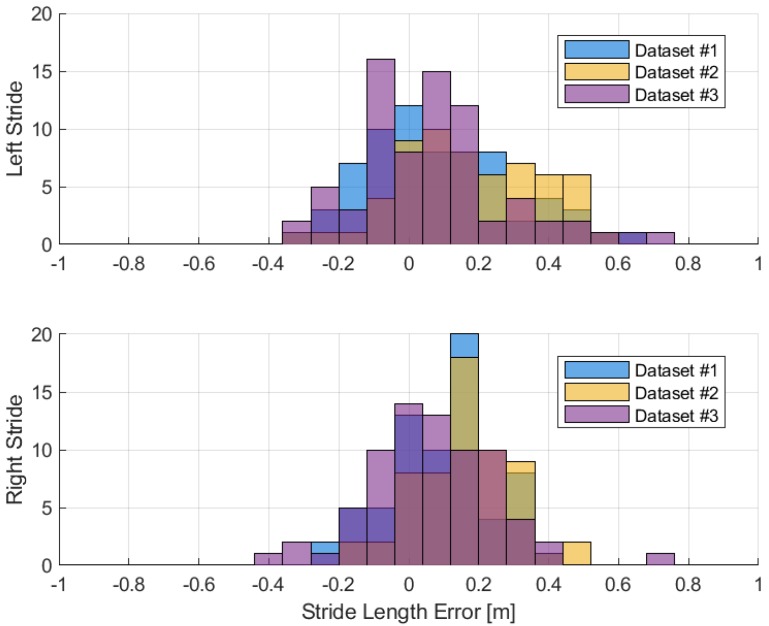
Foot stride length error.

In terms of stride length estimation, the foot strapdown algorithm also does not perform well. The distributions of stride length errors are shown in Figure 18 and Table 6. In general, this method has larger step size error and can lead to large positioning error due to the inertial sensor error accumulation. From these results, the performance of the strapdown algorithm depends on the quality of the inertial sensor used and the low-cost sensor is definitely not suitable for standalone positioning. By comparing the results of foot strapdown algorithm and the foot stride length of forward kinematics, it is better to use multiple low-cost sensors combined with gait kinematics in estimating the stride lengths rather than a single foot sensor.

## 5. Discussion

This research investigates the feasibility of a pedestrian navigation system utilizing multiple low-cost inertial sensors distributed on the lower limbs. A sensor data logger is developed to record motion data from two sensor types: MPU-6050 and MPU-9250. Seven sensors are used to record the motions of lower limb segments during a treadmill walk. A five-segment skeletal model along with its forward kinematics is developed to estimate step sizes. Different step size estimation methods are presented and compared to a reference solution obtained from an optical motion capture system.

Many of the past works have only used a single higher-cost inertial sensor. The higher-cost sensors tend to have less measurement error. The works on foot-mounted sensors by Foxlin [11] and Jiménez et al. [14] used the strapdown algorithm to track the position of the foot in indoor environments. Foxlin [11] reported a positioning error of 36 cm after walking a 118.5 m path inside a wood-frame house, while Jiménez et al. [14] reported a positioning error of 37.5 cm after walking a 125 m path inside a campus building. This kind of result indicates the strapdown algorithm can estimate the foot position as well as the heading with great accuracy. Jiménez et al. [14] have shown that the strapdown algorithm accuracy depends heavily on the heading update rate and the correction quality.

When the strapdown algorithm is applied to our foot-mounted sensor, the mean error of the estimated stride length approaches a quarter of the reported foot positioning error (∼40 cm) and the heading error is not yet accounted for. This shows that the strapdown algorithm is not suitable for such low-cost sensors. Looking into the strapdown algorithm, the sensor measurements (specific forces and angular rates) go through multiple integral functions in order to compute the position solution. The positioning error growth is proportional to cubic time for gyroscope error and proportional to squared time for accelerometer error.

In addition, the statistics of Table 5 clearly show the problem of using a mathematical model-based algorithm in estimating the stride length. Dataset #2 is used as the training data for estimating the tuning parameter *k* such that the mean error of this dataset is always the lowest. The results of the other two datasets show that the tuning parameter does not fit well and a similar trend happens with the mathematical model-based step length estimation.

Recently, Lee et al. [30] attempted to integrate the left and right foot positions computed using the strapdown algorithm with the heading and position solution computed from a pelvis-mounted sensor. In [30], multiple commercially available sensors from Xsens were distributed on the lower limbs. A kinematic model was used to link the pelvis and the two feet. The outdoor test results reported show that 5 m position error was achieved after walking a 400 m path, demonstrating that the use of kinematics of lower limbs can improve the positioning solution of a pedestrian navigation system.

By using distributed low-cost sensors and a skeletal model of lower limbs, the computation strategy changes from mechanizing the inertial equations, which are error-prone, to estimating each segment orientation. The position solution can be computed by using the forward kinematics of lower limbs based on the chosen skeletal model. According to Figure 10 and Table 3, the pelvis-mounted sensor can actually generate a descent heading estimate, although there is a small mean error of 2 degrees. The pitch errors of the thighs and shanks are also relatively small. The resultant step lengths have a mean error of 3.2 cm. The left stride length mean error is 12.5 cm while the right stride length mean error is 9 cm. The expected positioning accuracy is less than 5% of the total travelled distance.

## 6. Conclusions

While this paper is not able to show the complete performance of a pedestrian navigation system, acceptable step size and heading estimates are still achievable using multiple low-cost sensors. Foot gait detection can still be performed by this type of sensor. Segment orientation can be estimated with an accuracy of less than 6 degrees and using gait kinematics has shown that it can achieve a step length accuracy of 3.2 cm. This study shows that a wearable multi-sensor system combined with kinematics of the lower limbs can be used as an alternative to a single sensor in a pedestrian step-heading navigation system. However, the results presented, using a treadmill, do not assess the ability of the forward kinematics to detect changes in walking direction. Additional tests involving straight paths and turns are required.

Future work includes redesigning the sensor data logger for higher data rates and investigating the system performance with walking experiments in real environments. With these low-cost sensors, a wearable multi-sensor system embedded in clothing and human daily activity monitoring are realizable. This system could also be expanded for athlete performance monitoring such as running, skating, or cycling.

## Figures and Tables

**Table 1:** Specifications of MPU-6050 and MPU-9250.

Parameters	MPU-6050	MPU-9250
Accel. full-scale range	±2 g, ±4 g, ±8 g, ±16 g	
Accel. non-linearity	0.5%	0.5%
Accel. cross-axis sensitivity	±2%	±2%
Accel. noise power spectral density	400 μg/$\sqrt{\mathrm{Hz}}$	300 μg/$\sqrt{\mathrm{Hz}}$
Gyro. full-scale range	±250°/s, ±500°/s, ±1000°/s, ±2000°/s	
Gyro. Non-linearity	0.2%	0.1%
Gyro. cross-axis sensitivity	±2%	±2%
Gyro. rate noise spectral density	0.005°/s/$\sqrt{\mathrm{Hz}}$	0.01°/s/$\sqrt{\mathrm{Hz}}$
Gyro. total RMS noise	0.05°/s-rms	0.1°/s-rms
Gyro. initial zero tolerance	±20°/s at 25 °C	±30°/s at 25 °C
Magnetometer full-scale range	−	±4800 μT

**Table 2:** Results of swing phase detections.

Dataset	Swing Leg	Optical Motion Capture		Estimated Number of Swings	
		Number of Swings	Swing Frequency	Algorithm 1	Equation (1)
#1	Left	70	0.82 Hz	70	72
	Right	70		70	70
#2	Left	62	0.82 Hz	62	63
	Right	62		62	62
#3	Left	76	0.83 Hz	76	78
	Right	76		75	76

**Table 3:** Statistics of estimated orientation errors.

Segment Angle	Mean (Degrees)	Std. Dev. (Degrees)	Maximum (Degrees)
Pelvis Roll	0.19	1.47	3.75
Pelvis Pitch	0.94	0.95	3.26
Pelvis Yaw	1.97	1.66	5.64
Left Thigh Pitch	5.57	4.22	17.74
Right Thigh Pitch	3.98	3.54	14.94
Left Shank Pitch	4.13	2.80	11.11
Right Shank Pitch	0.82	2.68	9.22

**Table 4:** Step length error statistics.

Dataset	Method	Mean Error (m)	Std. Deviation (m)	Maximum Error (m)
#1	Weinberg	0.051	0.052	0.181
	Left Hip Kinematics	0.085	0.038	0.259
	Right Hip Kinematics	0.086	0.039	0.277
	Foot Kinematics	0.024	0.027	0.089
#2	Weinberg	0.009	0.058	0.171
	Left Hip Kinematics	0.031	0.040	0.122
	Right Hip Kinematics	0.032	0.041	0.130
	Foot Kinematics	0.022	0.043	0.168
#3	Weinberg	0.042	0.046	0.131
	Left Hip Kinematics	0.085	0.031	0.167
	Right Hip Kinematics	0.086	0.030	0.178
	Foot Kinematics	0.032	0.028	0.103

**Table 5:** Stride length error statistics.

Dataset	Method	Left Stride Error (m)			Right Stride Error (m)		
		Mean	Std. Dev.	Maximum	Mean	Std. Dev.	Maximum
#1	Kim et al.	0.115	0.116	0.420	0.112	0.094	0.341
	Kinematics	0.125	0.061	0.305	0.068	0.035	0.138
#2	Kim et al.	0.016	0.098	0.250	0.002	0.072	0.166
	Kinematics	0.013	0.061	0.179	0.025	0.065	0.166
#3	Kim et al.	0.089	0.126	0.472	0.058	0.072	0.224
	Kinematics	0.123	0.072	0.268	0.090	0.035	0.170

**Table 6:** Error statistics of foot stride length computed by strapdown algorithm.

Dataset	Left Stride Error (m)			Right Stride Error (m)		
	Mean	Std. Dev.	Maximum	Mean	Std. Dev.	Maximum
#1	0.079	0.213	0.626	0.093	0.149	0.414
#2	0.172	0.204	0.579	0.161	0.136	0.464
#3	0.067	0.210	0.686	0.067	0.184	0.721
